# Moving beyond pain: the relationship between physical activity and physical self-concept among young women with endometriosis

**DOI:** 10.3389/fspor.2025.1494460

**Published:** 2025-01-23

**Authors:** Samantha Coquinos, Alexandre Oboeuf, Damien Vitiello

**Affiliations:** Institut des Sciences du Sport Santé de Paris (I3SP), URP 3625, UFR STAPS, Université Paris Cité, Paris, France

**Keywords:** endometriosis, physical activity, physical self-concept, mental well-being, quality of life

## Abstract

**Background:**

Endometriosis is a painful non-curable disease that affects women's quality of life, reducing their self-esteem and consequently their mental well-being. However, enhancing the physical self-concept could induce the development of the self-esteem. A suitable method to increase the physical self-concept would be physical activity (PA). The aim of this study was to evaluate the relationship between PA and physical self-concept in women with endometriosis under 30 years old and to evaluate whether the type of PA was associated with different physical self-concept scores.

**Materials and methods:**

A total of 198 women under 30 diagnosed with endometriosis responded to a survey. Physical self-concept was assessed using the short version of the *physical self-description questionnaire*. Women were also asked to answer to questions about their PA habits. The type of PA practiced was then assigned to 1 of the 3 following groups: relaxing activities (*n* = 14), activities without necessary interactions with other participants (*n* = 58), and activities including necessary interactions with other participants (*n* = 46).

**Results:**

Women under 30 years old participating in a regular PA (more than once a week) (*n* = 137) had a significantly higher physical self-concept than women not participating in a regular PA (*n* = 61) (*p* < 0.05). Results differed in physical self-concept sub-scales. The type of PA did not seem to make a difference in physical self-concept scores (*p* > 0.05).

**Conclusion:**

Young women with endometriosis should be advised to participate to a regular PA to increase their physical self-concept and therefore cope better with their anxiety and stress. All types of activities appear to be just as relevant for developing physical self-concept in young women with endometriosis.

## Introduction

Endometriosis affects about 1 woman out of 10 of reproductive age^1^. Despite a high prevalence, this disease is still misunderstood and difficult to treat (1–3). Lesions of ectopic endometrium evolve outside the uterus cavity, creating a systemic inflammation. Because of its dependence to estrogen, it is a chronic inflammatory disease. Despite a long diagnosis delay (4), symptoms can appear at a young age (e.g., just after the menarche). Symptoms vary greatly between women (1, 5). Indeed, some can have chronical pain, when others have cyclic pain, and some are asymptomatic. However, pain would not be related to the severity of the disease (6, 7), which means that the disease itself is not sufficient to understand its impact on women's life. As it is a chronical condition, endometriosis is often associated to a lower quality of life and an increase in anxiety and depression (8–10), especially in women experiencing pain (11).

The chronical condition of endometriosis may also affect the perception the women may have of their bodies. The literature reports that women with endometriosis perceive their body as a machine that has lost its functionality, no longer able to do what it was designed to do (12–14). This specific condition is also associated to a feeling of losing control of their body. As it is a chronic estrogen-dependent disease (1–3), it means that the body and symptoms vary within the month, and every month unpredictably (15). Finally, these perceptions may lead women to feel diminished by endometriosis and unattractive due to the shame and embarrassment associated with their symptomatic bodies (14).

The way we perceive our body would be closely related to physical self-concept (16, 17). The physical self-concept is a sub-domain of the global self-concept as described by Shavelson et al. (18). It is defined as the perception or evaluation of a person's physical abilities and physical appearance (16). Fox (19) explains that since the body is the interface between the individual and the world through its appearance, attributes, and abilities, physical self-concept plays a unique role in self-concept. Therefore, enhancing the physical self-concept could induce the development of the self-esteem (16, 20). The physical self-concept is particular as it depends on the internal judgment of individual abilities, but also on external criteria as these individual abilities are compared to those of a frame of reference. Depending on the social environment in which a person interacts, the internal perception of their physical abilities will be strongly influenced by the people to whom they compare. It has been shown that a lower self-esteem is related to a higher risk of anxiety, whereas a greater self-esteem helps to cope with life events and is related to an increase of mental well-being (21, 22). As for women with endometriosis, they tend to have a negative image of their body that can be associated to a lower quality of life (23). In such context, it should be interesting to determine how to improve their physical self-concept.

Among other things, physical activity (PA) seems to be a suitable method to improve the quality of life by enhancing self-perception (24). In addition, some studies described that there would be a reciprocal relationship between physical self-concept and participation in PA (25, 26). Indeed, PA can help individuals to achieve a positive self-image and promote psychological well-being, particularly through improved physical perceptions and body satisfaction (27). However, if PA has an influence on self-esteem, this influence might not be distributed homogeneously according to the types of physical practices (28–31). We cannot consider that yoga, tennis or volleyball have the same influence on self-esteem. Some can be played alone, others require the participation of other players, and they can be played in such different types of environments. Parlebas (31) created a classification that uses the unpredictability of the environment and the presence of other players in the game (partner/opponent) to categorize sports. In total, he created 8 categories. According to him, the fact that the environment might be changing or not during a game, and that the activity is played with or without partner(s) and/or opponent(s) is what creates different motor patterns. For example, sports in a setting without any changes in the environment and without any other players involved usually tend to stereotype motor patterns with high performance. If other players are involved, dynamic sociomotor patterns take place: there is a communication that creates uncertainty and modifies the behavior of the players (29, 32). In this context, holistic training which involves slow movements with high precision (e.g., yoga or pilates) may has a different correlation with physical self-concept in women with endometriosis as it requires a high personal physical perception compared to sports played individually against an opponent where the comparison is direct.

Thus, the present study aims to evaluate: (1) the relationship between PA and physical self-concept in women with endometriosis under 30 years old; (2) whether the type of PA is associated with different physical self-concept scores in that population. We make the following hypotheses: (i) women with endometriosis under 30 who participate in a regular PA have a higher physical self-concept than the ones not participating in any regular PA; (ii) women who regularly engage in physical activities with partners and/or opponents have a lower physical self-concept than those who participate in activities alone or in simple co-presence.

## Methods

### Participants

French women with endometriosis over 18 years old were asked to respond to a questionnaire over physical perception. Data was collected from a larger study that includes adult women of all ages. As this study aimed young women, eligibility criteria included women aged between 18 and 30 years old. As an early care could help reducing the risk of anxiety and depression and increase their quality of life, this study focused on young women under 30 years old.

A total of 198 participants completed the questionnaire, with a mean age of 25.9 years old. Among the 198 women, 137 stated practicing a regular PA (average year of diagnosis 2020) and 61 declared not practicing any regular PA (average year of diagnosis 2021). Sample description is displayed in Table 1.

**Table 1:** Participants presentation.

	Number of respondents	Age, mean	Minimum age: maximum age	Average year of diagnosis
Regular PA	137	25.9 years old	18: 30	2020
No regular PA	61	25.8 years old	19: 30	2021

### Procedure

Participants recruitment was carried out using different means:

- Flyers with a QR code redirecting to the online questionnaire were displayed in different structures specialized in French endometriosis care, physiotherapy clinics, doctor clinics…;
- Through social media posts on endometriosis related groups;
- Sharing the questionnaire link via EndoFrance Newsletter.

The social media posts and the flyer stated that the research team was inviting participants to complete an anonymous questionnaire over PA and body perceptions of women with endometriosis and provided either a link or a QR code to access the questionnaire.

### Questionnaire

A small introduction explained to the participants what the aim of this questionnaire was, specified that the answers were strictly anonymous and confidential and that it took about 15 min to fulfill it.

Then, participants had to complete the short form of the physical self-description questionnaire (PSDQ-S). The original long version was validated and translated into French (33), and consists of 70 items rated on a 6-point Likert scale but takes time to answer. We chose the short form of 40 items for two reasons. First, Maïano et al. (34) described this short French version of the PSDQ as having acceptable psychometric properties. They specify that this questionnaire can be used with complete confidence in research in French. The overall minimum score is 40 and the maximum score is 240: the higher the score is, the better is the physical self-concept. The short form of the questionnaire also divides the overall score into eleven specific dimensions: health (5 items, score range from 5 to 30), coordination (5 items, score range from 5 to 30), physical activity (4 items, score range from 4 to 24), adiposity (3 items, score range from 3 to 18), sport competence (3 items, score range from 3 to 18), physical appearance (3 items, score range from 3 to 18), strength (3 items, score range from 3 to 18), flexibility (3 items, score range from 3 to 18), endurance (3 items, score range from 3 to 18), overall physical satisfaction (3 items, score range from 3 to 18) and finally, overall self-esteem (5 items, score range from 5 to 30). Second, as we asked additional questions about their PA habits and demographic characteristics, it seemed more accurate to use the short form to reduce the overall length of the questionnaire. Indeed, the next part of the questionnaire asked about their PA habits, the type of PA practiced, the duration and frequence of PA, and the amount of time that the respondent had been participating a regular PA. The last part of the questionnaire asked about some demographic and health related characteristics with the participant's age, year of diagnosis, occupation, and endometriosis related treatment.

Ethics approval was granted from the French committee of people's protection South-Ouest and overseas^2^, with the Paris Saint Joseph Hospital as the reference center.

### Data analysis

To evaluate the relationship between PA and physical self-concept among women with endometriosis, physical self-concept and subscales scores were compared between women practicing a regular PA, that was defined by at least once a week, and women not practicing any regular PA. Data between the two groups was analyzed with a comparison of means (+/− standard deviation). Variables distribution was assessed. Variables respecting a normal distribution were compared using a Student's *T*-test and the effect size using Cohen's D. The others were compared using Mann-Whitney's *U*-test and the effect size using a rank biserial correlation.

For the secondary analysis which aims to evaluate whether the type of PA is associated with different physical self-concept scores, we decided to draw inspiration from Parlebas (31) classification of PA. This classification includes 8 categories. Considering the number of respondents, the closeness of some categories and the aim of this study, we decided to classify the physical activities as the following:

1. Holistic training (pilates and yoga);
2. Physical activities performed without the direct influence of other players (running, fitness, swimming, etc.);
3. Sport performed with the direct influence of other players (partners/opponents: tennis, dance, judo…).

For this secondary analysis, participants are described in Table 2. Some of the responders did not fulfill the question asking about the type of PA they participated to. From the 137 responders who declared participating in a regular PA, a total of 118 participants has been considered for the data analysis on the type of PA.

**Table 2:** Respondents participating in a regular PA classified in PA type.

	Number of respondents	Age, mean	Minimum age: maximum age
Holictic training	14	25.9 years old	21: 30
PA without direct influence	58	25.6 years old	18: 30
PA with direct influence	46	26.3 years old	18: 30

A one-way ANOVA was used to compare the physical self-concept scores between women with endometriosis participating in different types of physical activities.

The software Jamovi (version 2.5) was used to perform the statistical analyses.

## Results

### Physical self-concept

Among the 198 women with endometriosis who completed the questionnaire, 137 declared participating in a regular PA, defined as at least once a week, and 61 did not. There was a significant difference in physical self-concept scores between women participating in a regular PA (*n* = 137, *M* = 134.56, SD = 23.61) and women not participating in a regular PA (*n* = 61, *M* = 108.05, SD = 21.25), *p* < 0.001. The effect size, measured by Cohen's *d*, was *d* = 1.157, indicating a large effect.

For the subscales, scores were significantly higher in the group participating in a regular PA (*n* = 137) compared to the group that did not (*n* = 61) for the subscales coordination (*p* < 0.001, *r*_rb_ = 0.49), physical activity (*p* < 0.001, *r*_rb_ = 0.83), sport competence (*p* < 0.001, *r*_rb_ = 0.57), physical appearance (*p* < 0.05, *r*_rb_ = 0.19), strength (*p* < 0.001, *r*_rb_ = 0.53), flexibility (*p* < 0.001, *r*_rb_ = 0.45); endurance (*p* < 0.001, *r*_rb_ = 0.52), and overall physical satisfaction (*p* < 0.001, *r*_rb_ = 0.57). The scores for the sub-scales health and adiposity were significantly higher in the group without regular PA (*n* = 61) compared to the group with regular PA (*n* = 137) (heath: *p* = 0.002, *r*_rb_ = 0.28; adiposity: *p* = 0.024, *r*_rb_ = 0.20). The sub-scale Global esteem did not reveal any statistical difference between the groups (*p* = 0.81). Results are described in Table 3.

**Table 3:** Correlation between regular PA and PSDQ-S scores.

Scores	Group	N	Mean	Standard deviation	Test used	Value	*P* value	Effect size
Total	Regular PA	137	134.56	23.61	Student's *T*-test	7.518	<0.001	1.157
	No regular PA	61	108.05	21.25				
Health	Regular PA	137	16.58	6.29	Mann-Whitney *U* test	3,027	0.002	0.2757
	No regular PA	61	19.97	6.91				
Coordination	Regular PA	137	19.87	5.43	Mann-Whitney *U* test	2,135	<0.001	0.4892
	No regular PA	61	14.59	5.53				
Physical activity	Regular PA	137	13.84	5.44	Mann-Whitney *U* test	702	<0.001	0.8320
	No regular PA	61	5.87	2.67				
Adiposity	Regular PA	137	9.45	5.52	Mann-Whitney *U* test	3,342	0.024	0.2003
	No regular PA	61	11.39	5.70				
Sport competence	Regular PA	137	10.38	3.65	Mann-Whitney *U* test	1,785	<0.001	0.5729
	No regular PA	61	6.46	3.65				
Physical appearance	Regular PA	137	10.36	3.41	Mann-Whitney *U* test	3,395	0.035	0.1876
	No regular PA	61	9.21	3.84				
Strength	Regular PA	137	9.57	3.36	Mann-Whitney *U* test	1,972	<0.001	0.5281
	No regular PA	61	6.38	3.13				
Flexibility	Regular PA	137	10.28	3.97	Mann-Whitney *U* test	2,299	<0.001	0.4499
	No regular PA	61	7.23	3.34				
Endurance	Regular PA	137	7.64	4.00	Mann-Whitney *U* test	1,989	<0.001	0.5240
	No regular PA	61	4.36	2.04				
Overall physical satisfaction	Regular PA	137	9.80	3.68	Mann-Whitney *U* test	1,799	<0.001	0.5695
	No regular PA	61	6.11	3.11				
Overall self-esteem	Regular PA	137	16.80	2.65	Mann-Whitney *U* test	4,090	0.811	0.0212
	No regular PA	61	16.48	2.98				

### Physical self-concept depending on the type of sport

Among the women practicing a regular PA (*n* = 137), 118 specified the sport(s) they practiced regularly. If they did more than one sport, the PA practiced most regularly was used to assign the answer to a group. The three categories were: (1) holistic training (*n* = 14, *M* = 129.21, SD = 21.80); (2) physical activities performed without the direct influence of other players (*n* = 58, *M* = 139.48, SD = 26.73); (3) sport performed with the influence of other players (*n* = 46, *M* = 133.78, SD = 20.51). A one-way ANOVA revealed that there was not a statistically significant difference in physical self-concept scores between the groups [F(2,115) = 1.37, *p* = 0.257]. Results are shown in Table 4.

**Table 4:** Correlation between the different type of PA and PSDQ-S total score.

Scores	Group	*N*	Mean	Standard deviation	F value	*P* value
Total	Holistic training	14	129.21	21.80	1.3745	0.257
	No influence of other player(s)	58	139.48	26.73		
	Direct influence of other player(s)	46	133.78	20.51		

## Discussion

The aim of this study was to evaluate the relationship between PA and physical self-concept in women with endometriosis under 30 years old and to evaluate whether the type of PA was associated with different physical self-concept scores. Endometriosis is a complex disease that is not exclusively related to the pelvis (35, 36). European Society of Human Reproduction and Embryology (ESHRE) recommendations (37) indicates that clinicians should consider the presence of shoulder pain and chest pain in the diagnosis of endometriosis, highlighting the need to address the disease as a whole and treating all chronic and systemic symptoms (35). In this approach, physical and psychological factors appear to be crucial to improve long term physical and mental health among women with endometriosis (38). This systemic approach raises the question of the positive influence of PA on the well-being of women with endometriosis. Yet, this population engage in less PA than women without this condition (39).

The results of the present study revealed that physical self-concept could be significantly increased among women with endometriosis by participating in a regular PA (*p* < 0.001). Participating in a regular PA could modify positively their body perception: they might gain confidence and realize that their body is still able to be functional and perform. It could modify positively their perception on their physical self and identity (24).

As for the sub-scales, most were significantly in favor for the group participating in a regular PA (strength *p* < 0.001; physical activity *p* < 0.001; endurance *p* < 0.001; sport competence *p* < 0.001; coordination *p* < 0.001; physical appearance *p* < 0.05; flexibility *p* < 0.001; overall physical satisfaction *p* < 0.001). As stated above, women with endometriosis tend to perceive negatively their body (12, 14, 15). Nevertheless, these results revealed that participating in a regular PA would be related to a better physical self-concept, and therefore, increase self-esteem (16, 20) and quality of life (24).

However, the scores for the sub-scales health and adiposity were significantly higher in the group without PA (*p* < 0.05), and the sub-scale overall self-esteem did not reveal any statistical differences between the groups (*p* > 0.05). The physical self-concept is dependent of internal and external criteria (17, 19, 40). Internal criteria are the descriptive aspect of an ability: it includes a person's knowledge and beliefs about themselves. But this description of an ability is associated to a self-assessment, correlated with external criteria: it is affected by the frame of reference. The fact that the physical self-concept can be affected by the group oneself compares to is called the *big fish little pond effect* (41), and this effect also takes place in PA (40). This means that independently of a person's level of PA, it is the level of the people one compares to, related to its environment, that will influence the physical self-concept.

The *big fish little pond effect* could explain why some sub-scales were lower in the group of women engaged in regular PA. If women with endometriosis participate in a regular PA, they might be surrounded by other healthy women to whom they compare to. Sayer-Jones and Sherman (14) explained that younger women with endometriosis described more frequent comparison to healthy women and a greater body dissatisfaction than older women with endometriosis who struggled more with other problematics (e.g., sexual functioning, weight gain, and fertility). Moradi et al. (42) also found that endometriosis-related body image concerns differ across age groups. For young women affected by endometriosis, seeing healthy women not struggling as much with body changes and not feeling lowered cyclically during PA might decrease their physical perception on their health and body fat.

The results obtained here also suggest that all types of PA performed regularly are correlated to similar physical self-concept scores (*p* > 0.05). However, we considered three different classes of PA: holistic training (yoga, pilates) that involves a high physical perception; PA without a direct influence of other players; and PA with a direct influence of other player(s) involving more communications (28–31). Considering the *big fish little pond effect* (40), we could consider that involving other healthy player(s) would have harmed the physical self-concept. But the scores of physical self-concept did not differ significantly between the different types of PAs. Considering those results, it can be argued that for women with endometriosis, the increase of their internal perception because of PA, realizing that their body is still functional and efficient, is more important on their physical self-concept than the possible external negative comparison with healthy women.

This tendency is continuing as holistic training would have a similar correlation to physical self-concept in women with endometriosis. Pilates have been described to increase physical self-concept in adult women (43), and this association appears to be similar on women with endometriosis. The results of the present study are correlated to Gonçalves et al. (44) findings as they found that yoga practice would be associated with a reduction of chronic pelvic pain and an improvement of quality of life among women with endometriosis. Those activities (i.e., pilates, yoga) are focused on physical perception, controlling the body with precision. On the other hand, they don't involve direct evaluative methods as there is no timer or opponent. Once again, these results suggest that physical self-concept for women with endometriosis increases when internal physical perceptions are involved, despite the lack or presence of direct external comparison. Therefore, the influence of PA seems to not be necessarily correlated to the level of physical engagement and relaxation activities represent an interesting alternative to fight the negative effects of endometriosis on physical perceptions.

The reciprocal relationship between physical self-concept and PA has already been described in literature (25–27). The results of this study imply the existence of this reciprocal relationship between PA and physical self-concept among young women with endometriosis. As PA contributes to the development of the physical self-concept (19, 24–26), it subsequently increases one's ability to cope with life events and is related to an increase of mental well-being (21, 22), reducing the risk of anxiety and depression (8–10).

## Limitations

1. The number of respondents between groups were not equal and the group performing a regular holistic training only had 14 respondents. It can be explained by the fact that young women are not usually as attracted to those physical activities. For example, a study described the mean age of yoga participants to be 51.7 (±11.7) years old (45).
2. The questionnaire was anonymous and fulfilled without the supervision of any instructor. The participants' answers were not verified, and the diagnosis was self-reported.
3. Some responders participated in more than one sport. Therefore, classifying each respondent into one category does not consider the influence of the participation to multiple PA.
4. The cross-sectional design of this study impedes to infer causality. Detailed demographic and clinical data that could serve as confounders were not described.

## Conclusion

Higher physical self-concept scores appear to be associated to regular PA. Young women with endometriosis should be advised to participate in any regular physical activity that pleases them as a higher physical self-concept reduces the risk of anxiety and depression and therefore increases their self-esteem and quality of life. In the present study, the type of physical activity performed does not seem to be relevant. Further longitudinal or interventional studies are needed to explore causal pathways.

## Data Availability

The raw data supporting the conclusions of this article will be made available by the authors, without undue reservation.

## Footnotes

^1^ https://www.who.int/news-room/fact-sheets/detail/endometriosis

^2^National number: 2023-AO2041-44 ; Registration number : 23.03404.000226.
